# Perceived social support, perceived stress, and quality of sleep among COVID-19 patients in Iran: assessing measurement invariance of the multidimensional scale of perceived social support across gender and age

**DOI:** 10.3389/fpsyt.2024.1337317

**Published:** 2024-04-18

**Authors:** Mojtaba Habibi Asgarabad, Zahra Vahabi, Ho Nam Cheung, Reza Ahmadi, Samaneh Akbarpour, Mohammad Hossein Sadeghian, Farnaz Etesam

**Affiliations:** ^1^ Department of Psychology, Norwegian University of Science and Technology, Trondheim, Norway; ^2^ Department of Geriatric Medicine, Tehran University of Medical Sciences, Ziaeian Hospital, Tehran, Iran; ^3^ Cognitive Neurology and Neuropsychiatry Division, Department of Psychiatry, Tehran University of Medical Sciences, Roozbeh Hospital, Tehran, Iran; ^4^ Department of Social Work and Social Administration, The University of Hong Kong, Hong Kong, Hong Kong SAR, China; ^5^ Department of Clinical Psychology, School of Medicine, Shahid Beheshti University of Medical Sciences, Tehran, Iran; ^6^ Occupational Sleep Research Center, Baharloo Hospital, Tehran University of Medical Sciences, Tehran, Iran; ^7^ Department of Forensic Medicine, School of Medicine, Tehran University of Medical Sciences, Tehran, Iran; ^8^ Psychosomatic Medicine Research Center, Department of Psychiatry, Tehran University of Medical Sciences, Imam Khomeini Hospital Complex, Tehran, Iran

**Keywords:** perceived social support, COVID-19, perceived stress, sleep quality, psychometrics

## Abstract

**Background:**

Perceived social support (PSS) plays a considerable role in mental health. The Multidimensional Scale of Perceived Social Support (MSPSS) is one of the most widely used scales, leading to much research evidence. The present study investigated its measurement model, equivalence across gender (male and female) and age groups (older patients= above 60 and non-older patients= below 60), and concurrent validity.

**Methods:**

A cross-sectional survey was conducted between March and October 2020, on patients hospitalized due to COVID-19 in Tehran, Iran. The scales were administered to 328 COVID-19 patients (54.6% male, aged 21 to 92) from two general hospitals; participants completed MSPSS (including friends, family, and significant others subscales), Pittsburgh Sleep Quality Index (PSQI, include sleep latency, subjective sleep quality, habitual sleep efficiency, sleep duration, use of sleep medication, daytime dysfunction, and sleep disturbances subscales), and the Perceived Stress Scale-10 (PSS-10, to assess patients’ appraisal of stressful conditions).

**Results:**

The MSPSS three-factor structure was confirmed among COVID-19 patients by Confirmatory Factor Analysis (CFA). The results support the MSPSS internal consistency and configural, metric, and scalar invariance across gender and age groups. Nevertheless, small but significant differences were found across ages based on the latent factor mean of the MSPSS from friends, with a lower mean level in older patients. The coefficients of Cronbach’s alpha (ranging from.92 to.96), the ordinal theta (ranging from.95 to.98), and Omega (ranging from.93 to.97) suggested high internal consistency of MSPSS. The concurrent validity of MSPSS was evidenced by its significant negative correlation with PSS-10 (τ_b_ = -.13, p <.01) and also subjective sleep quality (τ_b_ = -.22, p <.01), sleep disturbances (τ_b_ = -.26, p <.001), and daytime dysfunction (τ_b_ = -.26, p <.001).

**Conclusions:**

The MSPSS was valid and reliable for measuring individuals’ perception of social support between males and females and older and non-older COVID-19 patients.

## 1 Introduction

Since December 2019, the coronavirus outbreak and its variants have spread worldwide (1–3). Coronavirus Disease 2019 (COVID-19) patients report significant psychiatric and psychological sequelae, including anxiety, stress, depression, posttraumatic stress disorder, cognitive deficits, loneliness, and sleep disturbances (4–8). However, patients’ mental problems varied according to their perception of social support (9). Perceived social support (PSS), which describes how people evaluate and believe about the capacity of their social network and resources against stress and crisis, has recently been considered as a protective factor during the COVID-19 period (10, 11). PSS refers to the extent to which people find family members, friends, and other significant people in their lives available for support (12). The more these people are present in times of need and provide emotional, practical support and practical solutions, the more empowered a person feels to face problems. However, in the absence of social support, people feel lonely and isolated (13). Lack of PSS is associated with some psychological problems and difficulties in effectively coping with crises. PSS is essential among COVID-19 patients who were faced with social restrictions such as preventing them from meeting their relatives. Previous evidence has shown that lack of social support can predict high levels of stress (14) and sleep problems (15), which play a significant role in immune system weakness and lack of recovery (16).

Coping with stress and its psychological consequences is difficult without PSS (17). Evidence has shown that having social support during the COVID-19 crisis was effective in facilitating the management of related stress (18). PSS can reduce stress levels (19), enhance coping strategies in response to stressful conditions (20), and protect individuals against the physical and psychological consequences of adverse life events (21). However, when the patients feel lower level of PSS, they are more prone to non-adherence to medication (22).

PSS is associated with several aspects of sleep quality (23). During the COVID-19 pandemic, people with less PSS reported significantly more sleep problems than others (10). When people feel they have social support, they experience a greater sense of security, belonging, and relaxation, which affects their sleep quality (24). However, the lack of PSS is associated with loneliness, anxiety and depression, aggravating sleep problems (10, 25).

Mixed evidence has been reported on the difference in perceived social support across genders. Some studies have shown that perceived social support does not differ by gender (26, 27). In contrast, some research has revealed that gender affects the quality and quantity of perceived social support (28, 29). For example, depressed males received more social support from their friends, while depressed females received more support from significant others in their lives (30). Additionally, among cancer patients, males perceived less social support than female peers (31).

Similarly, mixed evidence was drawn from previous studies regarding the age differences in perceived social support. Some studies have shown that age does not create differences in the level of perceived social support and its components (32, 33), while F Li, S Luo, W Mu, et al. (34) examined the social support that individuals receive from different sources during COVID-19, and indicated the association between getting older and high perceived social support from sources outside of the family. On the contrary, a psychometric study of MSPSS reported a significant opposite trend that younger patients (56-65 years) perceived more outstanding social support from significant others than older patients (above 66 years).

The Multidimensional Scale of Perceived Social Support (MSPSS) is a valid instrument that assesses PSS in all three domains of family, friends, and significant others. The factor structure and psychometric properties of the MSPSS have been analyzed in different populations (**Table 1**). For example, investigations of the MSPSS among general populations have indicated that MSPSS three-factor structure is reliable (58). In a clinical population of cancer patients in Spain, it is shown that the original three-factor structure has a good model fit and substantial measurement invariance in gender and age (40). Similar results were also reported among Korean non-metastatic breast cancer survivors (aged 31–73) (35). However, the MSPSS’s factor structure and construct validity are not yet well known among COVID -19 patients.

**Table 1:** Studies validating psychometric properties of the MSPSS in different countries and populations.

Country, Year	Article	Participants	Ethnicity/race	Factor structure	Reliability (Alpha)
Korea, **2022**	(35)	N = 349, Female breast cancer survivors; M_age_ = 50.95	Korean	CFA-3	Ranged= .90 to.96
USA, **2022**	(36)	N = 242, Resettled Burmese refugees, Male= 53.3%, M_age_= 34.45	Burmese	EFA-3	Ranged= .94 to.96
China, **2021**	(37)	N= 487, Parents of children with CP, Mothers =366, Fathers= 121; M_age_ = 33.69	Chinese	CFA-3; EFA-2 (family and non-family)	Ranged= .87 to.89
Pakistan, **2021**	(38)	N= 1154, Pregnant women, 570 with depression, 584 without depression, M_age_ = 26.69	Pakistani	CFA-3	Ranged= .92 to.96
Romania, **2021**	(39)	N = 282, Romanian elite athletes, Male= 62.4%, M_age_= 21	Romani	CFA-3	Ranged= .90 to.91
Spain, **2021**	(40)	N = 925, Patients with cancer, Male= 39.7%, M_age_= 59	Spanish	CFA-3	Ranged= .93 to.94
Indonesia, **2020**	(41)	N = 299, Adolescent survivors of a volcanic eruption, Male= 47.8%, M_age_= 15.02	Indonesian	CFA-3	Ranged= .74 to.83
USA, **2019**	(42)	N = 303, Inpatient and out-patient participants who were recruited from cardiology centers, Male= 63%, M_age_= 21	Caucasian= 65% Other= 35%	CFA-3	Ranged= .92 to.95
Greece, **2019**	(43)	N = 150, Nurses (80 mental health nurses and 70 oncology nurses), Male = 49%, Age= 36 to 45	Greek	CFA-3	Ranged= .95 to.96
USA, **2018**	(44)	N= 223, First-year college students, 14 to 18 years, Male = 36%, M_age_ = 18.60	Hispanic or Latino	CFA-3	Ranged= .91 to.94
Colombia, **2018**	(45)	N= 766, Schooled adolescents, Male= 55.2%, M_age_ = 15.7	Colombian	CFA-3	Ranged= .75 to.84
Italy, **2018**	(46)	N= 236, Patients with chronic disease, 14 to 18 years, Male= 55.2%, Mage = 15.7	Italian	CFA-3	Ranged= .92 to.96
USA, **2017**	(47)	N= 475, patients with confirmed heart failure, Male = 31%, Age = 61 ± 12	White= 72% Non-White= 28%	KMO-3	All three factor are similar= .94
Zimbabwe, **2017**	(48)	N= 120, Caregivers to outpatient patients with cancer, Female= 69.2%, M_age_ = 59.6	Zimbabwean	EFA-2, CFA-2 (Friends and family/significant others)	Ranged= .84 to.89
USA, **2015**	(49)	N = 122, Undocumented Hispanic immigrants, Male= 57.37%, M_age_ = 33.66	Hispanic	PCA-2 (friends and family/significant others)	Total = .93
Iran, **2013**	(50)	N = 176, Patients with consecutive myocardial infarction, Male= 84%, M_age_ = 56; 71 healthy participants, Male= 65%, M_age_ = 74.4	Iranian	EFA-3	Patients sample ranged= .85 to.93 Healthy sample ranged= .87 to.92
Sweden	(51)	N = 282, Including 127 women with hirsutism, M_age_= 30; 154 nursing students, Male = 11.7; M_age_ = 23.5	Swedish	EFA-3	Ranged= .93 to.95
China, **2012**	(52)	N= 301, South Asian migrants in Hong Kong (Pakistani and Nepalese); 153 Nepalese, Male (47.7%), M_age_= 33.2; 148 Pakistani, Male (39.2%), M_age_= 32.4	Pakistani- Urdu= 49.17% Nepalese= 50.83%	Nepalese, EFA-3 Pakistani, EFA-2 (family and significant others)	Nepalese, Ranged= .80 to.86 Pakistani, Ranged= .90 to.91
Thailand, **2011**	(53)	N= 462, 310 medical students, Male (41%), M_age_= 19.16; 152 psychiatric outpatients who had a history of diagnosis of major depressive disorder, Female (55%), M_age_= 41.23	Chinese	CFA-3	Students, Ranged= .83 to.91 Patients, Ranged= .74 to.85
USA, **2010**	(54)	N = 539, Arab immigrant women residing in the United States, Mage = 40.2	Iraqi= 43.8% Lebanese= 33.6% Yemeni= 13.4% Other Arab countries= 10.2%	CFA-3	Ranged= .73 to.89
Pakistan, **2010**	(55)	N=325, Antenatal women, Age ranged= 17-40	Pakistani with Urdu language	PCA-1	Total = .92
Turkish, **2008**	(56)	N= 433, School administrators, Male (50.5%), Age= 90% of participant were above 40 years	Turkish	CFA-3	Ranged= .87 to.92
USA, **1988**	(57)	N= 275, University undergraduates, Male (50.5%), M_age_= 18.6	–	CFA-3	Ranged= .85 to.91

CFA-3, confirmatory factor analysis- three factors (friends, family, and significant others); EFA-3, exploratory factor analysis- three factors (friends, family, and significant others); CFA-2, confirmatory factor analysis- two factors; EFA-2, exploratory factor analysis- two factors; KMO-3, Kaiser Meyer Olkin-three factors (friends, family, and significant others); PCA-1, principal components analysis- single factor; PCA-2, principal components analysis- two factors.

As a developing country in the Middle East, Iran has made significant progress in its healthcare system. However, it was severely affected by the outbreak of the Covid-19 pandemic. Culturally, Iranian people have a collective culture, and family relationships are still the main focus of social support for individuals. However, in critical situations, including illness, individuals receive various social supports from other sources. Even if the COVID-19 pandemic terminates, we risk an outbreak of coronaviruses (59) or other potential pandemics (60). Therefore, examining the three-factor model and measurement invariance of PSS in COVID-19 patients and the relationship of its factors with stress and sleep quality as two highly correlated factors can expand our knowledge about the reliability and validity of the PSS model. It can also show which of the PSS sources are more related to stress and reduced sleep quality in patients so that related interventions can be considered. Evidence related to it can help the medical service system to improve the quality of patient care and treatment during hospitalization and recovery. Consequently, the present study was conducted to investigate the three-factor structure of the MSPSS, test for gender and age invariance, and estimate psychometric properties such as internal consistency and concurrent validity among COVID-19 patients. Therefore, we identified the following hypotheses based on previous evidence:

- The MSSS among COVID-19 patients will follow the same three-factor structure (family, friends, and significant others) as the original version.
- Gender invariance in the MSPSS factor structure will be equal in the configural, metric, and scalar invariance.
- Age invariance in the MSPSS factor structure will be equal in the configural, metric, and scalar invariance.
- The invariance of latent factor means will be similar by gender and age groups on MSPSS scores from friends, family, and significant other.
- The MSPSS will have acceptable internal consistency based on Cronbach’s alpha.
- The MSSS will have acceptable concurrent validity based on its negative correlation with sleep problems.
- The MSSS will have acceptable concurrent validity based on its negative correlation with perceived stress.

## 2 Methods

### 2.1 Participants

The 328 hospitalized COVID-19 patients (54.9% male) aged 21 to 92 (M = 50.77, SD = 15.32) were selected from two general hospitals (Baharloo and Ziaeian Hospitals) with a hospitalization duration of 7 days, on average. The inclusion criteria consist a history of hospitalization due to COVID-19 and the exclusion criteria include a) lack of interest in participating in the study, b) insufficient ability to answer questions, and c) the patient’s death after discharge from the hospital. Most participants had diploma education (78, 23.2%) and were housewives (n= 119, 36.3%). In addition, patients mostly lived with their spouses and children (n= 209, 63.7%) and had no smoking history (n= 284, 86.6%). The demographic characteristic of the sample is presented in **Table 2**.

**Table 2:** The description of the sample characteristics (n = 328).

Age, years, M ± SD		50.77 ± 15.32
Age group, n (%)		
below 60 years		89 (27.1%)
above 60 years		239 (72.9)
Gender, n (%)		
Female, n (%)		146 (44.5)
Male, n (%)		179 (54.6)
Living, n (%)		
alone		34 (10.4)
with spouse		50 (15.2)
with children		32 (9.8)
with spouse and children		209 (63.7)
Education, n (%)		
illiterate		63 (19.2)
elementary		75 (22.9)
secondary		54 (16.5)
diploma		78 (23.8)
advanced diploma or bachelor		46 (14)
Master or PhD		11 (3.4)
Patients’ Job, n (%)		
employee (managers)		13 (4)
employee (non-managers)		39 (11.9)
labor		20 (6.1)
housewife		119 (36.3)
unemployed		10 (3)
private		67 (20.4)
retired		43 (13.1)
others		77 (23.5)
Family members, n (%)		
one		25 (7.6)
two		68 (20.7)
three		77 (23.5)
four		114 (34.8)
five		35 (10.7)
six		4 (1.2)
ten		2 (0.6)
Role in family, n (%)		
independent		24 (7.3)
family head		120 (36.6)
dependent		97 (29.6)
Ability to use medications independently, n (%)		
yes		67 (20.4)
no		25 (7.6)
Ability to do housework, n (%)		
yes		61 (18.6)
no		13 (4)
Psychiatric history, n (%)		
yes		15 (4.6)
no		313 (95.4(
Smoking categories, n (%)		
never		284 (86.6)
previous smoking		33 (10.1)
currently smoker		8 (2.4)
Smoking years, M ± SD		3.06 ± 8.67
Alcohol history, n (%)		
yes		14 (4.3)
no		308 (93.9)
Drugs abuse history, n (%)		
positive		7 (2.1)
negative		317 (96.6)
Duration of hospitalization, days, M ± SD		7.11 ± 5.97

### 2.2 Recruiting, assessing, and conducting procedure

The current cross-sectional study conducted between March and October 2020, on patients hospitalized due to Coronavirus infection at Baharloo and Ziaeian Hospitals in Tehran, Iran. After confirming the positive diagnosis of coronavirus, demographic information of patients, including gender, age, educational and occupational status, and their contact numbers, were collected in a registration form.

Participants read and assented to the informed consent form before starting the study. Then, three psychology experts collected data (the MSPSS, the PSS-10, and the PSQI) via telephonic interview from those who filled in the consent form. The implementation of the measurement phase was carried out by measurement experts familiar with the tools. Before starting the assessment, participants received instructions on how to answer the questions. It was also explained to them that if any of the items were unclear, they should discuss it with their evaluator. If the person had a wrong understanding of the items, the necessary explanations were provided. This study was approved by the ethical board of Tehran University of Medical Sciences (Ethic Code: IR.TUMS.VCR.REC.1399.156).

### 2.3 Measurements

#### 2.3.1 The multidimensional scale of perceived social support – Persian version

The MSPSS (57) has a 12-item, developed to measure how people think about their social support assets (including friends, family, and significant others). In addition, it has a 7-point Likert scale, ranging from 1 (*strongly disagree*) to 7 (*strongly agree*), scored between 12 to 84, where higher scores on this scale mean greater perceived social support. Psychometric studies examining the MSPSS in different populations supported the original three-factor solution (39, 43, 52, 57). Furthermore, some studies that examined MSPSS in the patient population while confirming its three-factor structure have shown good psychometric properties (61).

#### 2.3.2 Perceived stress scale – Persian version

The PSS (14 items-version) was presented to evaluate patients’ appraisal of stressful conditions in their life (62). Subsequently, the developers endorsed a 10-item version of the tool (63). This 10-item unidimensional scale comprised four positively and six negatively worded items. Items were scored through a 5-point Likert scale, between 0 (*never*) to 4 (*very often*), where positively worded items were reversely coded. The original study of PSS-10 reported good internal consistency (α= .78) (63). Moreover, the Persian version of PSS-10 also demonstrated high internal consistency reliability (α= .93) (64).

### 2.3.3 Pittsburgh sleep quality index – Persian version

The PSQI (65) is a self-administered 19-item tool for a brief assessment of sleep disturbances that influence sleep quality within the prior month. The seven components of PSQI include sleep latency, subjective sleep quality, habitual sleep efficiency, sleep duration, use of sleep medication, daytime dysfunction, and sleep disturbances). PSQI is scored on a 4-point Likert scale, and the components’ scores are counted together to obtain the total sleep quality score. The total PSQI score ranged between 0 and 21, with high scores showing poor quality of sleep and the cut-off point of higher than 5 for identifying poor sleepers. A good internal consistency was reported for the original PSQI (Cronbach’s α= .83) in the preliminary study (65), as well as the internal consistency of PSQI (α= .81) was acceptable among the Iranian sample (66).

### 2.4 Statistical strategy

The Confirmatory factor analysis (CFA) with less bias and a more robust maximum likelihood (MLR) estimator was adopted to examine *a priori* models of the factor structure by HN Cheung and MJ Power (67). Statistical strategies were as follows: First, as it is recommended for ordinal Likert-type scales, the internal consistency was examined using Cronbach’s alpha, mean inter-item correlation, and the equivalent of Cronbach’s alpha coefficients (Theta and Omega), which are based on the polychoric correlation, rather than the Pearson correlation (68, 69) and was conducted in R version 4.1.2 (70, 71). According to a rule of thumb, a correlation coefficient of.70 or higher was considered an acceptable level of internal consistency of the items (72).

Second, we used the following statistical tests and indices (73–78) to evaluate the fitness the models (parenthesis indicate acceptable values): the chi-square (*χ^2^*; p >.05), the Tucker–Lewis index (TLI >.95), the comparative fit index (CFI >.95), the normal chi-square (3 > *χ^2^*/df < 2), the standardized root mean square residual (SRMR <.05), the root mean square error of approximation (RMSEA <.05), and the confidence interval of 90%. A low Bayesian information criterion (BIC) points to a good model fit. The comparison of competing models was carried out using a chi-square difference test. A nested model is more restrictive than a baseline model since it has more degrees of freedom (79).

Third, having selected the most appropriate model, we tested its measurement of gender equality. Invariance of factorial structure/pattern (configural invariance), corresponding factor loadings (metric invariance), and finally, corresponding indicator means (scalar invariance) were evaluated. We tested the differences in RMSEA, SRMR, and CFI of nested models for invariance (80–82).

Finally, the concurrent validity was determined by examining the correlation between the MSPSS and PSQI, as well as PSS-10, using Kendall’s coefficient (τ_b_) for non-normal data. Correlations in this study were interpreted as having effect sizes of small (.10), medium (.30), large (.50), and very large (.70) (83).

## 3 Results

### 3.1 Factor structure

**Figure 1:**
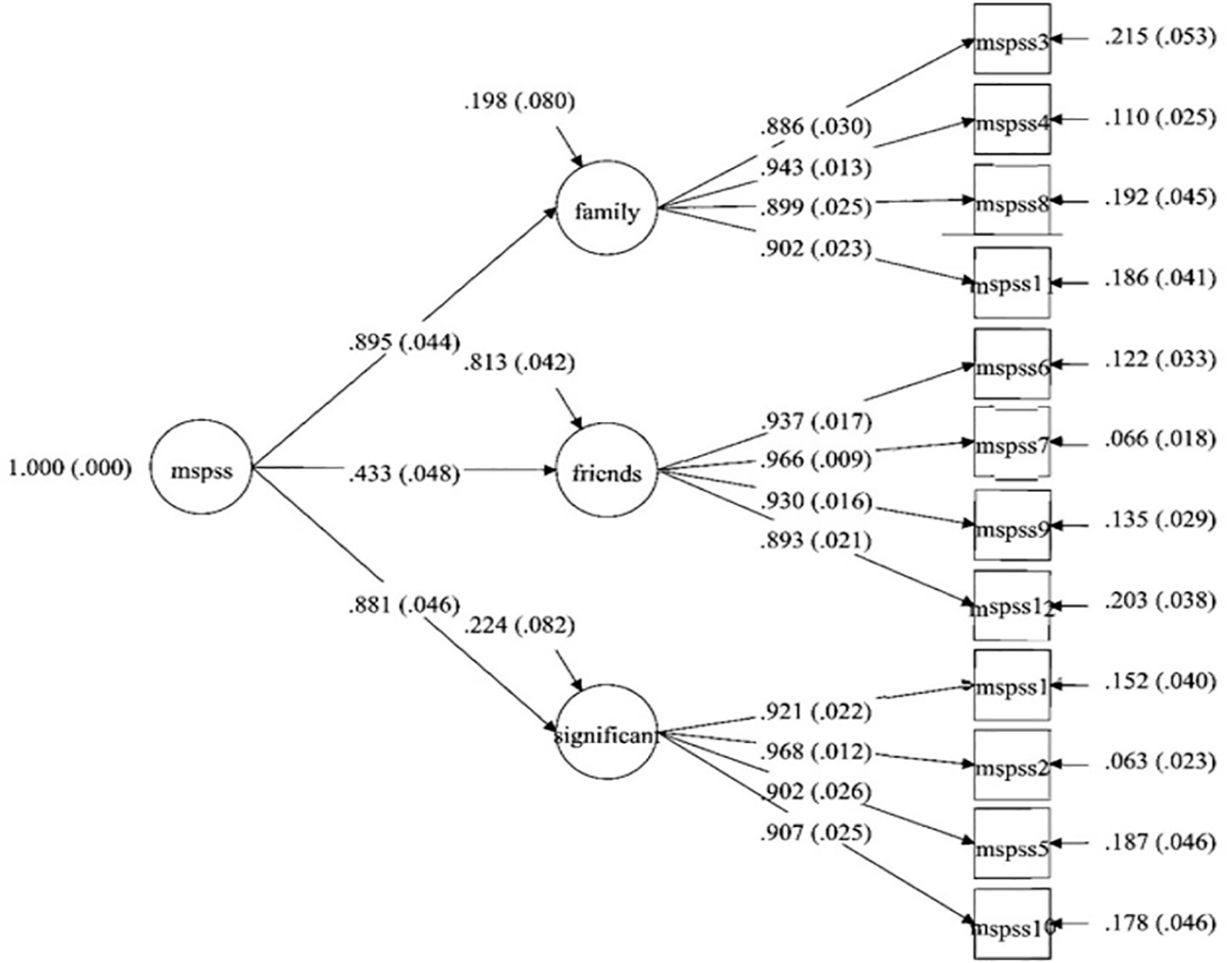
The three first-order and one second-order confirmatory factor analysis of the MSPSS.

The Mplus 8.7 version was utilized to ascertain the MSPSS factorial validity (84), and the goodness of fit was tested for four models. The first model (M1) examined a general solution in which items were loaded over a general social support component to test the unidimensional model of assumed latent factor and included just random measurement error and indicator-specific variance (85). If the general factor model fitted well with the data, the assumption of the multidimensionality of the measurement tool was violated. In other words, it could be interpreted as the lack of discrimination validity for subscales of psychological tools. Model 2 (M_2_) consisted of a three-factor orthogonal model containing three uncorrelated latent factors. According to the literature, model 3 (M3) examined a three-factor oblique model resembling the exploratory factor analysis (67). Eventually, model 4 (M4) encompassed a three-factor first-order loaded on the one-factor second-order. In the higher-order model, more than one orthogonal first-order subordinate factors mediate the relationship between observed indicators and superordinate second-order latent factor (86). Based upon the largely standardized covariances among the latent factors in M3, a second-order model may be needed to account for the estimated variances and covariances of three perceived social support subscales (**Figure 1**).

### 3.2 Model selection

As indicated in **Table 3**, oblique three-factor model (M_3_) and the three-factor first-order and one-factor second-order model’s fit indices (M_4_) met most of the specified criteria. They also yielded better model fit, compared to nested models (M_1_ and M_2_). Then, the parsimonious principle (87) was used to compare the M4 fit indices with those of the M3 as competitive models. The results indicate that based on the fitness indices, neither of these independent models has any distinct advantages (see **Table 3**), although the three-factor first-order and the one-factor second-order model can be optimized/parsimonious based on the theory-derived model.

**Table 3:** Means, standard deviations, internal consistency coefficients, fit indices for CFA of MSPSS.

Subscale	Item number	M	SD	SK	KU	r^cs^	r^ct^	CAID	OTID	Ordinal Theta	Omega	M_1_	M_2_	M_3_	M_4_	Male (Female)		Below than 60 (Above than 60)	
Significant others	1.	5.36	1.61	-1.95	.025	.89	.73	.95	.97	.97	.96	.89	.92	.92	.92	.90 (.95)		.90 (.97)	
	2.	5.43	1.57	-1.30	.365	.93	.77	.94	.98			.94	.97	.97	.97	.96 (.97)		.97 (.98)	
	5.	5.50	1.50	-1.45	.949	.88	.70	.95	.95			.88	.90	.90	.90	.91 (.91)		.89 (.91)	
	10.	5.43	1.52	-1.44	.782	.89	.74	.95	.93			.90	.91	.91	.91	.93 (.89)		.88 (.97)	
Family	3.	5.61	1.40	-1.65	1.87	.85	.71	.94	.93	.97	.95	.78	.89	.89	.87	.86 (.91)		.87 (.91)	
	4.	5.57	1.39	-1.57	1.58	.91	.68	.92	.97			.82	.95	.94	.94	.92 (.96)		.95 (.93)	
	8.	5.41	1.47	-1.40	.791	.87	.68	.94	.96			.78	.90	.90	.90	.95 (.85)		.90 (.90)	
	11.	5.50	1.40	-1.58	-1.49	.87	.73	.93	.97			.81	.90	.90	.90	.89 (.92)		.88 (.96)	
Friends	6.	4.12	1.98	.007	-1.61	.90	.66	.95	.98	.98	.96	.45	.94	.94	.94	.96 (.91)		.94 (.94)	
	7.	4.16	1.98	-.036	-1.61	.94	.77	.94	.97			.43	.97	.97	.97	.97 (.97)		.97 (.97)	
	9.	4.29	1.99	-.145	-1.63	.92	.76	.95	.97			.42	.93	.93	.93	.93 (.93)		.94 (.91)	
	12.	4.33	1.98	-.18	-1.62	.88	.63	.96	.97			.40	.89	.89	.89	.87 (.91)		.90 (.97)	
Model	*χ^2^*	*df*	*χ^2^*/df	CFI	TLI	BIC		RMSEA		SRMR	Base Model		Δ*χ^2^* (df)			ΔCFI	ΔTLI	ΔRMSEA	ΔRMR
M_1_	954.11	54	17.67	.526	.421	12477.39		.225 (.213-.238)		.189	–		–			–	–	–	-
M_2_	267.69	54	4.96	.888	.863	10778.37		.110 (.097-.123)		.343	M_1_		686.42 (51)^***^			.362	.442	-.115	.154
M_3_	78.89	51	1.55	.985	.981	10460.95		.041 (.022-.058)		.023	M_1_		219.64 (3)^***^			.459	.56	-.184	-.166
M_4_	78.89	51	1.55	.985	.981	10460.95		.041 (.022-.058)		.023	M_1_		219.64 (3)^***^			.459	.56	-.184	-.166
Measurement invariance across gender																			
males	99.68	51	1.95	.957	.944	5779.23		.073 (.051-.094)		.026	–		–			–	–	–	-
females	84.02	51	1.65	.963	.952	4785.78		.066 (.039-.091)		.043	–		–			–	–	–	-
Configural	183.38	102	1.80	.960	.948	10619.45		.070 (.053-.086)		.035	M_4_		107.343 (51)***			-.025	-.033	.029	.012
Metric	192.48	111	1.73	.960	.952	10579.85		.067 (.051-.083)		.047	Configural		8.333 (9)			.000	.004	-.003	.012
Scalar	199.28	120	1.66	.961	.957	10529.15		.063 (.047-.079)		.047	Metric		1.428 (9)			.001	.005	-.004	.000
Measurement invariance across age (above and below 60 years old people)																			
Below 60	71.46	51	1.40	.985	.980	7690.87		.041 (.013-.062)		.030	–		–			–	–	–	-
Above 60	116.53	51	2.28	.913	.888	2839.80		.120 (.091-.149)		.035	–		–			–	–	–	-
Configural	178.84	102	1.75	.965	.954	10593.88		.068 (.051-.084)		.031	M_4_		104.729 (51)***			-.02	-.027	.027	.008
Metric	192.94	111	1.74	.963	.955	10558.34		.067 (.051-.083)		.044	Configural		13.658 (9)			-.002	.001	-.001	.013

M, Mean; SD, standard deviation; SK, skewness; KU, kurtosis; r^cs^, corrected item-total correlation for subscales` items; r^ct^, corrected item-total correlation for total scale’s items CAID, Cronbach’s alpha if item deleted; OTID, Ordinal Theta if item deleted; M_1_, factor loadings in the general one-factor for 12 items; M_2_, factor loadings in the three-factor orthogonal model; M_3_, factor loadings in the three-factor oblique model; and M_4_, factor loadings in the three-factor first-order and one-factor second order model; χ^2^, Chi-square; df, degrees of freedom; TLI, Tucker–Lewis index; CFI, comparative fit index; BIC, sample-size adjusted Bayesian information criterion; χ^2^/df, normal chi-square; Δχ^2^, Difference between minus twice log likelihoods between the full and the nested models; Δdf, difference in degrees of freedom between the full and nested models; SRMR, standardized root mean square residual; RMSEA, root mean square error of approximation; Δ, differences between parameters of two models; χ^2^, significant χ^2^ change indicates non-invariance of the model that hierarchically was compared with the previously ordered model. ^∗∗∗^p <.001.

In examining gender invariance (males and females) and age invariance (above 60 years old (older patients) vs. below 60 years old (non- older patients)), a multi-group CFA analysis was performed in the total sample, males and females groups, and also in older and non-older groups to obtain a satisfactory fitness for the baseline model in each one, under the parsimony and meaningfulness principle (88). Then, configural, weak, and strong measurement invariance were evaluated (**Table 3**) (81, 89, 90).

Given that changes in the model fit index were minimal, configural invariance was established for the M_4_ across gender (male vs. female) and age groups (older vs. non-older patients). Comparison of metric model with configural model (*ΔCFI* = .000, *ΔTLI* = .005, *ΔRMSEA* = .003, *ΔRMR = .0012*), scalar model with metric model (*ΔCFI* = .001, *ΔTLI* = .005, *ΔRMSEA* = .004, *ΔRMR = .000*), as alternative models indicated that three-factor oblique model (**Table 3**) was invariant across gender.

Similarly across age groups, comparison of metric model with configural model (*ΔCFI* = .002, *ΔTLI* = .003, *ΔRMSEA* = .003, *ΔRMR = .0012*), scalar model with metric model (*ΔCFI* = .001, *ΔTLI* = .005, *ΔRMSEA* = .002, *ΔRMR = .001*), as alternative models indicated that three-factor oblique model was invariant across age (**Table 3**).

### 3.3 Internal consistency

The descriptives, Cronbach’s alpha, Theta (ordinal alpha), Omega reliability coefficients, and corrected item-total correlation for the MSPSS subscales are presented in **Table 3**. The MSPSS Cronbach’s α was.94, and for friends, family, and significant others was from.92 to.96, suggesting an excellent internal consistency. Almost all the items within the three subscales had a moderate positive relationship with each other-with values ranging from.85 to.94 (according to a corrected item-total correlation for items in each subscale), and from.63 to.77 (using item-total correlation corrected for the scale’s items). Finally, the means of inter-item correlation were.55,.86,.82, and.87 for the total score, significant others, family, and friends’ subscales, respectively.

### 3.4 Latent factor mean differences social support across gender and age

A testing invariance of latent factor means showed significant group similarity by gender and age on perceived social support scores from friends, family, and significant other (p >.05, **Table 4**). Nevertheless, the older patients scored significantly lower on the latent factor mean level of the friends’ subscale of perceived social support (mean differences = .47, z = 2.03, p <.05) than the non-older patients.

**Table 4:** The correlation coefficients of MSPSS, PSQI subscales and PSS, Means, Standard Deviations and Latent mean differences.

	1	2	3	PSQI							PSS	Mean (SD)			Mean (SD)		Latent mean differences (Z)^a^	Latent mean differences (Z)^b^
				SSQ	SL	SDu	HSE	SDi	USM	DD		total	female	male	below 60 years	above 60 years		
1. Significant others	–	–	–	-.28^***^	-.08	-.17^*^	.07	-.24^***^	-.01	-.24^***^	-.09^*^	22.00 (5.78)	21.86 (5.88)	21.55 (5.86)	22.16 (5.69)	21.54 (6.02)	-.03 (-.24)	-.22 (-1.30)
2. Family	.75^**^	–	–	-.20^***^	-.06	-.11	.06	-.29^**^	-.05	-.27^***^	-.11^**^	17.04 (7.63)	17.17 (7.42)	16.64 (7.69)	17.46 (7.69)	15.86 (7.39)	-.09 (-.54)	-.21 (-1.10)
3. Friends	.35^**^	.36^**^	–	-.17^*^	-.09	-.07	.04	-.22^**^	.02	-.17^**^	-.14^**^	22.45 (5.07)	22.18 (5.18)	22.03 (5.42)	22.53 (5.05)	22.24 (5.14)	-.14 (-.65)	-.47 (-2.03)^*^
4. MSPSS	.83^***^	.82^***^	.77^***^	-.22^**^	-.10	-.10	.07	-.26^***^	-.02	-.26^***^	-.13^**^	61.49 (14.83)	60.94 (14.96)	61.81 (14.80)	62.16 (14.79)	59.65 (14.88)	–	–

SSQ, subjective sleep quality; SL, sleep latency; SDu, sleep duration; HSE, habitual sleep efficiency; SDi, sleep disturbances; USM, use of sleeping medication; DD, daytime dysfunction; a, latent mean differences across gender; b, latent mean differences across age groups; ∗p <.05, ∗∗p <.01, ∗∗∗p <.001.

### 3.5 Concurrent validity of MSPSS

**Table 4** showed the inter-correlation between MSPSS subscales. Correlation coefficients ranged from.35 to.83. Concurrent validity was estimated by the testing correlation of total MSPSS and its subscales with PSQI components and PSS-10 (**Table 4**). Kendall’s correlation coefficients showed total MSPSS has a negative significant correlation with subjective sleep quality (τ_b_ = -.22, p <.01), sleep disturbances (τ_b_ = -.26, p <.001) and daytime dysfunction (τ_b_ = -.26, p <.001). Furthermore, MSPSS has significant negative correlations with PSS-10 (τ_b_ = -.13, p <.01).

## 4 Discussion

This study aimed to evaluate the MSPSS measurement model, its equivalence in terms of gender and age, and concurrent validity in patients discharged from COVID-19 inpatient care. Both the three-factor first-order and one-factor second-order models had adequate fit indices to the data. All items were loaded on corresponding factors according to the three-factor first-order and one-factor second-order models. These results signify that the most acceptable model to expound on the MSPSS would be considering the three-factor (family, friends, and significant other). In this regard, the results of the present study are consistent with previous literature (35, 57) concerning the three-factor structure of MSPSS among COVID-19 patients, rather than the one-factor model (55).

Due to the test for gender and age invariance in the MSPSS factor structure, results illustrated equalities in the configural, metric, and scalar invariance (81, 91). In other words, the structure of the MSPSS measures the same construct by gender and age groups, which is in line with former studies in other samples (40, 92). Based on the latent factor mean differences, it can be concluded that males and females with COVID-19 perceive the social support of their resources similarly. From these data, it can be concluded that females and males probably have similar needs for social support due to the history of hospitalization due to COVID-19, and they interpret and understand it in the same way. Likewise, older and non-older patients perceive social support from two sources, family and significant others. However, when it comes to social support for friends, older patients perceive less social support than non-older patients. Probably, during the period of the disease, due to the need for quarantine and isolation, patients have less contact with friends than family and important people in life, and older patients are more likely than younger people to comply with social restrictions (93, 94).

Another principal aim of the study involved testing associations between perceived social support, sleep quality, and perceived stress. Perceived social support has been shown to correlate negatively with perceived stress among university students (95). In addition, it moderates the stress impact on mothers of sick children (96), and alleviates pain by reducing the stress of irritable bowel syndrome (IBS) patients, which refers to the social support stress-buffering role (28, 97). The results showed that perceived social support and perceived stress are negatively associated. These findings support the assumption that higher levels of social support can act as a protective factor against stress and highlight the importance of social relationships in modulating the stress of patients’ lives.

More specifically, as predicted, a significant negative relationship was found between perceived social support and sleep quality (subjective sleep quality, sleep disturbances, daytime dysfunction), in line with earlier evidence (98). The association between perceived social support and sleep quality is more influenced by its protective and moderating role on sleep quality risk factors. For instance, I Grey, et al. (10), demonstrated the association of perceived social support with better sleep quality during the COVID-19 pandemic, possibly due to the association of perceived social support with depression, irritability, and loneliness. In addition, R Xu, Y Lin and B Zhang (99) have shown that perceived social support can moderate the sleep quality’s relationship with the subjective well-being of older patients by buffering the effect of negative emotions. Overall, the perceived social support relationship with sleep quality seems to be affected by the impact of social support on feelings of loneliness, reduced adverse psychological reactions, and improved health status.

### 4.1 limitations, and future directions

Although the present study added considerable evidence to the literature, there were still limitations. Due to the unique circumstances of the outbreak of COVID-19, the voluntary sampling method was chosen for the present study. However, this method may make it difficult to access a complete representative sample of the community, which in turn can hinder the ability to generalize the results widely. This study included only COVID-19 patients over 21 years old. Therefore, caution should be exerted to generalize these findings to other populations and the age group under 21 years, as this study was conducted with people above 21 years old. In addition, only self-report data were contained in the present study; hence, associations between the variables may have been affected by shared method variance. Further, as this was a cross-sectional study, the causal relationship between perceived social support and other variables was not considered. Consequently, the results of the study may have been affected by some external factors such as seasons, the peak of the pandemic, and quarantine measures.

Despite that no difference was found in the factor structure of MSPSS based on gender and age in the present study, future investigations need to investigate these discrepancies in various groups more because the evidence for age and gender differences is relatively contradictory. Eventually, the Persian version of the MSPSS will be widely used in various clinical settings. Given Iran’s ethnic and cultural diversity, it is suggested that future studies examine the psychometric properties and effectiveness of this tool among Iranian different ethnic groups and cultures. Also, since coronaviruses and their various variants have been around in the past and are likely to reappear in the future and affect people’s lives, longitudinal research, multiple methods, and more specific disciplines are ought.

## 5 Conclusion

The MSPSS has an acceptable and promising factor structure, validity, reliability, and measurement invariance across gender and age among COVID-19 patients. This study provided support for the clinical utility of MSPSS in various populations. Given the high importance of perceived social for COVID-19 patients and people experiencing stressful events, MSPSS can be applied support to their perceived social support to look into the associations between sleep quality and perceived stress.

## Data availability statement

The raw data supporting the conclusions of this article will be made available by the authors, without undue reservation.

## Ethics statement

The studies involving humans were approved by Ethics Committee of Tehran University of Medical Sciences. The studies were conducted in accordance with the local legislation and institutional requirements. The participants provided their written informed consent to participate in this study.

## Author contributions

MHA: Writing – review & editing, Writing – original draft, Supervision, Software, Methodology, Funding acquisition, Formal analysis, Conceptualization. ZV: Writing – review & editing, Validation, Resources, Investigation, Data curation. HNC: Writing – review & editing. RA: Writing – review & editing, Writing – original draft, Visualization. SA: Writing – review & editing, Validation, Investigation, Data curation. MHS: Writing – review & editing, Visualization, Validation, Investigation. FE: Writing – review & editing, Writing – original draft, Resources, Project administration, Methodology, Funding acquisition, Conceptualization.
